# Dietary effects on resting metabolic rate in C57BL/6 mice are differentially detected by indirect (O_2_/CO_2_ respirometry) and direct calorimetry

**DOI:** 10.1016/j.molmet.2014.03.003

**Published:** 2014-03-21

**Authors:** Colin M.L. Burnett, Justin L. Grobe

**Affiliations:** Department of Pharmacology, The Obesity Research and Education Initiative, The Fraternal Order of Eagles' Diabetes Research Center, The François M. Abboud Cardiovascular Research Center, and The Center on Functional Genomics of Hypertension, University of Iowa, IA, USA

**Keywords:** Metabolism, Metabolic rate, Respirometry, Direct calorimetry, Energy, Obesity

## Abstract

Resting metabolic rate (RMR) studies frequently involve genetically-manipulated mice and high fat diets (HFD). We hypothesize that the use of inadequate methods impedes the identification of novel regulators of RMR. This idea was tested by simultaneously measuring RMR by direct calorimetry and respirometry in C57BL/6J mice fed chow, 45% HFD, and then returned to chow. Comparing results during chow feeding uncovered an underestimation of RMR by respirometry (0.010 ± 0.001 kcal/h, *P* < 0.05), which is equivalent in magnitude to ∼2% of total daily caloric turnover. RMR during 45% HFD feeding was increased by respirometry (+0.013 ± 0.003 kcal/h, *P* < 0.05), but not direct calorimetry (+0.001 ± 0.002 kcal/h). Both methods indicated that return to chow reduced RMR compared to HFD, though direct calorimetry indicated a reduction below the initial chow fed state (−0.019 ± 0.004 kcal/h versus baseline, *P* < 0.05) that was not detected by respirometry (−0.003 ± 0.002 kcal/h versus baseline). These results highlight method-specific interpretations of the effects of dietary interventions upon RMR in mice, and prompt the reevaluation of preclinical screening methods used to identify novel RMR modulators.

## 1 Introduction

Obesity is a major risk factor for the development of diabetes, cardiovascular disease, and cancer [1]. Current pharmacological therapies for obesity are limited to a very short list of compounds that function by suppressing food intake behavior or digestive efficiency. Understanding the mechanisms that control and regulate resting metabolic rate (RMR) may lead to the development of an adjunct or alternative approach to treating obesity.

Critical proof-of-concept evidence that stimulating RMR is an effective means to treat obesity comes from the storied history of 2,4-dinitrophenol (DNP), a mitochondrial ionophore that was banned for human use by the FDA (*ultimately due to unacceptable pharmacokinetics leading to overdosing and death*) in 1938 [2]. DNP worked to strongly induce RMR, and remains the most effective, yet unsafe, anti-obesity drug ever available. Because of its continued use in manufacturing processes, ill-informed and desperate consumers occasionally still obtain and use DNP for weight loss and body building purposes, frequently with tragically fatal consequences [3–6]. The quest to find safer but equally efficacious compounds relies upon sophisticated genetic and pharmacological techniques, but also upon technology to accurately and reliably assess the effects of such manipulations on RMR.

Respirometry (the most common type of “indirect calorimetry”) involves the estimation of metabolic rate of an animal based on the exchange of respiratory gasses with the animal's environment. This method is undoubtedly the dominant method used in modern metabolic research to assess RMR in mice [7–9], though the accuracy of respirometry for use in laboratory rodents (given its reliance on a series of untested/untestable assumptions) has been questioned [10,11]. Using a combined direct calorimeter/respirometer (“total calorimeter”) system, Walsberg and Hoffman demonstrated that respirometry variably and grossly underestimates RMR in various endotherms (kangaroo rat, dove, and quail) [12], but reliably estimates RMR in a specific ectotherm (python) [13]. We similarly used a total calorimeter to demonstrate that respirometry quantitatively underestimates the RMR of wildtype C57BL/6J and FVB/NCrl mice maintained on standard chow. Importantly, the large variability in rates of underestimation resulted in incorrect quantitative and qualitative conclusions as to the effects of ketamine/xylazine anesthesia in C57BL/6J mice, and genetic disruption of the angiotensin II type 2 receptor in FVB/NCrl mice [11]. We postulate that sole reliance upon respirometric methods may lead pharmaceutical researchers to inappropriately waste effort pursuing ultimately ineffective modulators of RMR, or to prematurely discard promising new targets and modulators. To test this hypothesis, we placed C57BL/6J mice on a sequential dietary program of chow, 45% high fat diet (HFD), then back to chow, and simultaneously measured RMR during each phase using both respirometry and direct calorimetry.

## 2 Materials and methods

### 2.1 Animals

Male C57BL/6J mice were obtained from the Jackson Laboratories at about 6–8 weeks of age. Throughout the study, mice were housed individually in forced-air shoebox style cages on a 12:12 light:dark cycle at 22 °C, with ad libitum access to food and water. At twelve weeks of age, mice underwent surgery under isoflurane anesthesia to implant core temperature telemeters (DSI, model TA-F10) in the abdominal cavity. At baseline, mice were maintained on a standard rodent chow (Teklad 7013; 18% kcal from fat). Starting at fifteen weeks of age, RMR was assessed weekly using a total calorimeter, as described below. Starting immediately after the recording session during the eighteenth week of age, mice were switched to a 45% HFD (OpenSource 12451; 45% kcal from fat). Immediately after the recording session, during the twentieth week of age, mice were switched back to standard chow. All studies were approved by the University of Iowa Institutional Animal Care and Use Committee.

### 2.2 Total calorimetry

Combined direct calorimetry and respirometry were performed using equipment, techniques, and calibration procedures recently described in detail [11] (summarized in Supplemental Methods). In contrast to our previous report, results obtained by direct calorimetry were empirically and individually corrected in the current study for core temperature changes. A direct calorimeter (custom-built through combined efforts of Heinz F. Poppendiek, PhD of Geoscience, Ltd. and our laboratory) was used to measure total heat dissipation (convective, conductive, evaporative, and radiative). Core body temperature was recorded by an antenna (DSI, pre-production ‘tethered antenna’) suspended from the ceiling of the calorimetry chamber. Core body temperature changes were used to calculate energy retention during the recording period, using the specific heat capacity of 0.874 kcal kg^−1^ K^−1^ (for a mouse with ∼10% body fat, interpolated from Blaxter [14]). NMR measurements (Bruker, model LF90) before implantation (week 12; 7.3 ± 0.8% fat, 76.8 ± 0.8% lean, 12.3 ± 0.2% fluid) and after removal (week 22; 10.7 ± 1.5% fat, 73.9 ± 1.1% lean, 12.2 ± 0.2% fluid) of radiotelemetric core temperature probes support the use of this value. The RMR assessed by direct calorimetry was therefore calculated by adding rates of heat retention to heat dissipation. Effluent air from the calorimetry chamber was continuously sampled for analysis of oxygen and carbon dioxide content. The respirometric RMR was estimated by gas exchange using the equation derived from Lusk [15], with *V*O_2_ measured in L/h and resultant *Heat* values in kcal/h:

$$Heat=V{\text{O}}_{2}\left(1.232\;RER+3.815\right)$$

### 2.3 Statistics

RMR results obtained by the two methods, and across feeding phases, were compared first by two-way repeated measures analysis of variance (RM ANOVA), followed by the all-pairwise Tukey's multiple-comparisons procedure.

## 3 Results

Mice exhibited expected, steady weight gains under baseline standard chow conditions (Supplemental Figure 1A). When switched to HFD, weight gains accelerated, and a return to standard chow conditions resulted in a return to the original weight-gain trajectory.

When the animals were maintained on standard chow, respirometry consistently underestimated RMR by 0.0101 ± 0.0008 kcal/h (6.85 ± 0.51%, *P* < 0.01 by paired *t*-test) (Figure 1A and B), as compared to results from direct calorimetry.

Upon the initiation of HFD feeding (weeks 19–20), the RMR measured by respirometry was indistinguishable from RMR measured by direct calorimetry (Figure 1A and B). Thus, RMR appears to significantly increase with the introduction of HFD when assessed using respirometry (+0.0129 ± 0.0031 kcal/h, *P* < 0.01). In contrast, direct calorimetry indicates that HFD has no effect on RMR (+0.0006 ± 0.0022 kcal/h) (Figure 1C).

Upon returning mice to standard chow (weeks 21–22), a significant reduction in RMR was determined by both methods, though the magnitude of the reduction was greater by direct calorimetry (Figure 1A and B). A 0.0090 ± 0.0025 kcal/h (5.93 ± 1.41%, *P* < 0.01) reduction in RMR was calculated by respirometry with the switch from HFD to chow, whereas a 0.0129 ± 0.0013 kcal/h (8.85 ± 0.89%, *P* < 0.01 versus HFD phase, and *P* < 0.01 versus respirometry) reduction was calculated by direct calorimetry. A return to baseline RMR values with the return to chow (+0.0039 ± 0.0020 kcal/h) was therefore indicated by respirometry, but a reduction compared to baseline chow RMR (−0.0122 ± 0.0022 kcal/h, *P* < 0.01 versus baseline, and *P* < 0.01 versus respirometry) was indicated by direct calorimetry (Figure 1C).

The magnitude of correction to direct calorimetry based upon heat retention was calculated for each animal during each recording session. Under baseline chow conditions and during the first week of HFD feeding, the core temperature was relatively stable during recording sessions, resulting in no significant net heat retention and therefore no correction to heat dissipation values. In contrast, beginning with the second week of HFD feeding (week 20), core temperature changes during recording sessions became progressively larger, and this resulted in very small corrections to RMR calculations (Figure 1D, Supplemental Figure 1C). Interestingly the lowest stable core temperatures achieved during sleeping bouts during the recording sessions (Supplemental Figure 1D) exhibited a slow increase as animals aged and gained body mass, but this steady increase was abruptly reversed with the switch from HFD back to standard chow.

## 4 Discussion

Previously, we determined that the baseline RMR of wildtype C57BL/6J and FVB/NCrl mice was underestimated when assessed by respirometry. Further, the variability of the underestimation by respirometry resulted in qualitatively incorrect conclusions with regard to the effects of specific pharmacological and genetic manipulations in these strains [11]. Here, we again show that respirometry underestimates the RMR of wildtype C57BL/6J mice maintained on a chow diet. The magnitude of underestimation (∼7%, 0.01 kcal/h, or 0.24 kcal/day) is the equivalent of approximately 2–3% of total daily caloric turnover in a mouse (∼10 kcal/day absorbed). This magnitude of underestimation by respirometry is similar in proportion to the changes in energy turnover that are necessary to elicit large, life-long changes in body mass and composition in humans [16,17], underscoring the need to improve upon the quantitative accuracy of simple respirometric methods for measuring RMR in mice.

Further, we demonstrate that when mice are fed a 45% HFD, the underestimation of RMR by respirometry is diminished such that respirometric and direct calorimetry methods yield indistinguishable RMR values. This results in increased quantitative accuracy of the RMR estimation by respirometry, but also the false suggestion that HFD feeding results in an increased RMR. Finally, we demonstrate that respirometry fails to accurately estimate the magnitude of RMR reduction when mice are switched from a 45% HFD back to standard chow. These findings have multiple major implications for the assessment of RMR in rodent models and the use of respirometry for these purposes.

First, these findings prompt a reconsideration of the ability of respirometry to accurately measure RMR in mice. As the relative accuracy of respirometry is variable (even within a single animal) and subject to manipulations such as diet or genetic background, there is no simple mathematical correction that can be universally applied to respirometry results [11]. This may be reflective of the inability of respirometry to detect energy turnover related to anaerobic and nitrogen cycling processes [10,12]. With increasing awareness of the importance of the (largely anaerobic) gut microbiome to whole-animal metabolic function [18, 19], it is tempting to hypothesize a role for shifts in anaerobic metabolism to explain the variability of accuracy of respirometry with dietary changes, for example. Whether real-time assessments of effluent methane, for example, could be used to correct future respirometric measures in mice remains to be empirically tested. Alternatively, it is likely that nitrogen turnover accounts for some fraction of the inaccuracy of O_2_/CO_2_ respirometry, and measurements of nitrogen intake and excretion are now considered a required additional step for respirometry-based studies [8,9]. It is important to recognize that measurements of nitrogen turnover require food, urine and fecal analyses, however, which results in a complete loss of the ability to distinguish nitrogen turnover during rest from nitrogen turnover during bouts of activity. Thus, nitrogen-based corrections to results from respirometry are appropriate for chronic, integrated, total energy expenditure analyses, but the utility of nitrogen turnover assessments to correct resting energy expenditure measurements (i.e. – RMR) is currently only a matter of speculation.

Second, and more importantly, these results underscore the inability to predict or even to identify post-hoc situations in which respirometry results accurately reflect RMR (e.g. – during 45% HFD feeding), underestimate RMR (e.g. – during baseline chow feeding), or overestimate RMR (e.g. – when returning to chow after two weeks on HFD). Thus, it is possible or perhaps even likely that some or many previous reports of the effects of various manipulations upon RMR may be qualitatively and/or quantitatively accurate, but the lingering concern is that it is impossible to know the accuracy of these findings without repeating key comparisons by direct calorimetry or other methods that are empirically demonstrated as reliably accurate.

We must consider the possibility that the differences in RMR results between methods could result from random or systematic errors in measurements by either or both methods. These types of problems, however, are unlikely for several reasons. First, careful calibration procedures were followed, and the high-quality equipment and procedures employed were chosen to ensure sufficient accuracy and precision (as outlined in the Supplemental Methods section). Second, empirical tests of the reliability of the direct calorimeter demonstrate very low variability (output voltage versus applied heat linear regression; slope = 0.0234 ± 0.0000403, and *R*^2^ value = 0.99992), and empirical tests of the respirometer setup similarly demonstrate extremely consistent results by this method (e.g. – ethanol burns [11]). Third, serial RMR measurements by each method remained steady over time (e.g. – Figure 1A, within the four weeks of baseline chow feeding). Fourth, random or even systematic errors in measurement within one or both methods are unlikely to explain the different patterns of intervention effects between methods. Switch onto HFD had a major, sustained effect by respirometry, but no effect was observed by direct calorimetry. Switch back to chow returned RMR to baseline values when assessed by respirometry, but an exaggerated effect was observed by direct calorimetry – and this exaggerated effect was also reflected in reduced core temperature values (Supplemental Figure 1D). Thus, we conclude that the differences between methods are a result of the essential differences in methods, as respirometry only measures the rate of carbon/oxygen-based aerobic processes while direct calorimetry reflects the sum of all metabolic processes, including anaerobic, nitrogenous, and carbon/oxygen-based aerobic functions.

Our choice of specific age (15–22 weeks), sex (male), strain (C57BL/6J), diets (an 18% kcal-from-fat chow, and a 45% HFD), and time-course (two weeks of HFD and two weeks following return to chow) prompts many questions as to the physiological ramifications of the data presented herein. Importantly, the purpose of this study was *not* to evaluate these *specific* manipulations upon the RMR of C57BL/6J mice, but rather to compare the accuracy and precision of respirometry with direct calorimetry, to assess the RMR effects of dietary manipulations. To this purpose, the current study design clearly illustrates the shortcomings of respirometry. Future studies, taking into consideration changes in body mass, body composition, sex, age, strain, ambient housing conditions, hydration choices and status, seasonal and diurnal rhythms, etc., are required to assess the RMR effects of specific interventions. The current data set provides strong evidence that such studies in mice should be performed using direct calorimetry instead of respirometry-based methods.

The primary complication of using direct calorimetry is the lack of commercial sources for this equipment. The equipment used in the current study was custom-fabricated by the authors and Heinz F. Poppendiek [11], but construction of such equipment is beyond the engineering prowess of many laboratories. Also, while the component costs and operating costs for gradient-layer calorimetry equipment are similar to high-quality continuous push–pull respirometry systems, the throughput of direct calorimetry is substantially lower than respirometric methods due to the need for simultaneous radiotelemetric core temperature recordings (which necessitates a survival surgery and thereby limits use to sufficiently-equipped laboratories and animal subjects of sufficient size and development to tolerate an implanted device within the abdomen). Ongoing development of alternative forms of direct calorimeters such as Peltier-style systems may provide a somewhat less-expensive method to measure heat dissipation [20], though the need for telemetric core temperature assessments remains. Studies utilizing direct calorimetry systems have been reported for preterm infant humans [21], but it is unclear whether this technology can be used with neonatal rodents for studies on developmental changes in metabolic function. Finally, systems that allow continuous 24-h metabolic rate measurements in rodents by direct calorimetry have apparently not yet been developed. The major hurdle to this development appears to involve the specific sequestration of urine and drinking water, as evaporating liquids confound real-time assessment of evaporative water loss by the test subject.

## 5 Conclusions

In summary, these results demonstrate as-yet unexplained differences in RMR results between direct calorimetry and respirometry, with regard to the effects of dietary interventions in mice. Acknowledging the many untested or untestable assumptions upon which respirometric methods rely, we hypothesize that these differences reflect the inability of respirometry to assess changes in anaerobic and nitrogen metabolism. Interpreting the data from this viewpoint, we conclude that respirometry incorrectly indicated a large induction of RMR with the introduction a 45% HFD (a false positive effect), and incorrectly indicated a return to baseline RMR values with the removal of the HFD (a false negative effect). These findings and this interpretation undermine confidence in studies investigating the effects of novel RMR-stimulating anti-obesity therapeutics that have only utilized respirometry-based methods. We posit that truly effective interventions might have been prematurely discarded for failing to cause inductions of RMR, when assessed only by respirometry (a consequence of false negative results). Further, the findings may explain the lack of efficacy of some promising new RMR-stimulating compounds when translating results from mice to humans, when efficacy in mice was only demonstrated by respirometry (a consequence of false positive results). We advocate the continued development and increased use of direct calorimetry for rodent studies to assess the efficacy of novel anti-obesity, RMR-stimulating therapeutics.

## Figures and Tables

**Figure:**
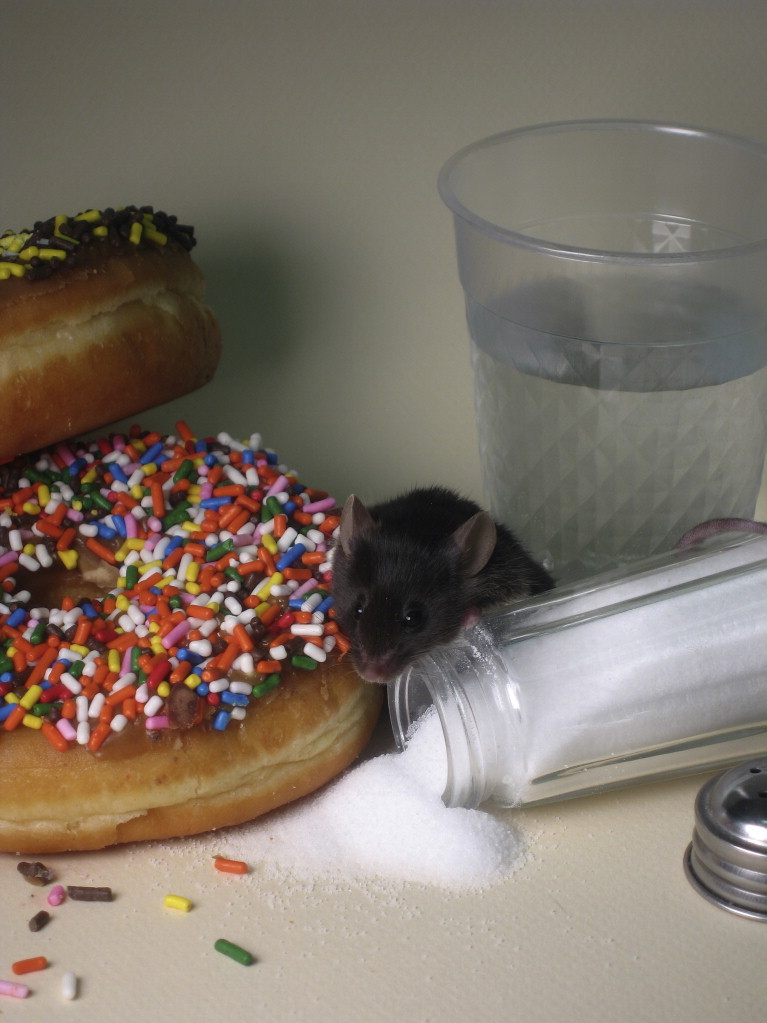


**Figure:**
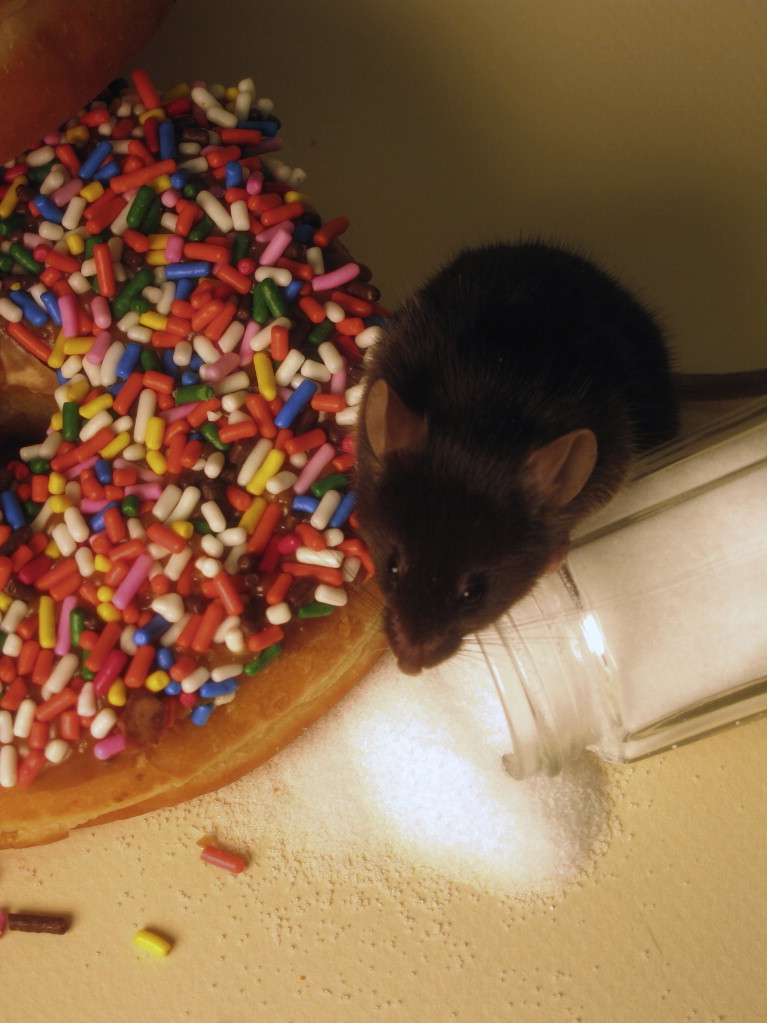


**Figure 1:**
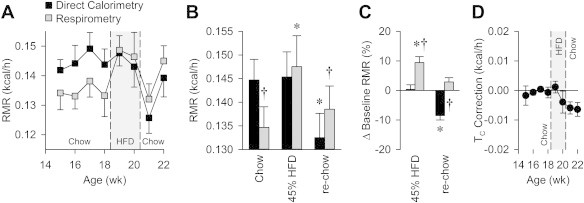
Effect of dietary interventions on RMR as simultaneously assessed using direct calorimetry and respirometry. (A) Heat production at thermoneutrality (30 °C) as simultaneously measured using direct calorimetry and respirometry. (B) Summary of data, averaged within subject during baseline chow (average of weeks 15–18), HFD (average of weeks 19–20), and the first week after reintroduction of chow (re-chow, average of weeks 21–22). (C) Summary of the effect of HFD and reintroduction of chow upon RMR, as compared to RMR measured during the baseline chow phase, illustrating the respirometry-based over- and under-estimation of the effects of dietary manipulations upon RMR. (D) Magnitude of correction to direct calorimetry results due to drift in core body temperature during recording sessions. A positive deflection indicates an increasing core temperature trend, while a negative deflection indicates a decreasing core temperature trend. For all panels, *n* = 5 male C57BL/6J mice. Data in panels B&C were analyzed by two-way repeated-measures ANOVA, followed by the Tukey's multiple-comparisons procedure. *P < 0.05 versus baseline chow phase within method. ^†^*P* < 0.05 between methods within diet phase. All data are mean ± SEM.
