# Supplementary material for: Dietary effects on resting metabolic rate in C57BL/6 mice are differentially detected by indirect (O2/CO2 respirometry) and direct calorimetry
Source: Mol Metab. 2014 Mar 21;3(4):460–4. doi: 10.1016/j.molmet.2014.03.003 (PMC4060218; doi:10.1016/j.molmet.2014.03.003)
Supplement: Supplementary file 1 [file mmc1.docx]

**Supplemental Data and Methods**

**Dietary effects on resting metabolic rate in C57BL/6 mice are differentially detected by indirect (O2/CO2 respirometry) and direct calorimetry**

Colin M.L. Burnett and Justin L. Grobe

*Department of Pharmacology, the Obesity Research and Education Initiative, the Fraternal Order of Eagles’ Diabetes Research Center, the François M. Abboud Cardiovascular Research Center, and the Center on Functional Genomics of Hypertension, University of Iowa*

Address correspondence to:

Justin L. Grobe, PhD, FAHA

Department of Pharmacology

51 Newton Rd., 2-307 BSB

Iowa City, IA 52242

Tel: (319) 353-5789

Fax: (319) 335-8930

Email: [justin-grobe@uiowa.edu](mailto:justin-grobe@uiowa.edu)

Supplemental Data





Supplemental Figure 1. (A) Body mass. (B) Respiratory exchange ratio. (C) Magnitude of correction factor to direct calorimetry resulting from drift in core temperature during the recording session. Data are the same as presented in Figure 1D, expressed as % of direct calorimetry result. (D) Lowest core temperature achieved during sleep.

Supplemental Methods

Full details on the construction, calibration, validation, and first use of our total calorimeter were previously published [[1](#_ENREF_1)]. A brief paraphrased summary follows.

*Direct Calorimeter*

A custom water-jacketed Seebeck-style direct calorimetry chamber was fabricated by Heinz F. Poppendiek, Ph.D., of Geoscience, Ltd, with a 10 x 10 x 10 cm interior chamber. Thermopile DC output was amplified 500x with DC high-pass and 5 Hz low-pass filtering (ETH-256, iWorks) before digitization (NI USB-6008, National Instruments). Influent and effluent gas enthalpy was measured using sensors (temperature/humidity - SHT15 Sensiron; pressure – BMP085, Bosch; flow – EM1NL1R0V, Sensiron) interfaced to a custom data acquisition program (programmed in LabView, National Instruments).

Heat dissipation (in kcal/hr) was calculated using the following equation [[2](#_ENREF_2), [3](#_ENREF_3)]:

$$Heat=Q_{w}+Q_{a}+Q_{m}$$

*Q_m_* denotes heat detected by the thermopile, *Q_a_* denotes heat of dry air, and *Q_w_* denotes heat carried by water vapor. *Q_m_* was calculated from thermopile output. Calibration was performed using a 10.97 Ω resistor, and a variable potentiometer to ensure linearity of the relationship between heat applied and calorimeter output voltage.

STP correction of flow rate (FRSTP) was performed:

$${FR}_{STP}=FR\times\frac{P}{101325}\times\frac{273.15}{T+273.15}$$

Where *FR* is the uncorrected flow rate (in mL/min), *P* is the air pressure (in Pa), and *T* is the air temperature (in °C).

*Q_a_* was determined by the following equation:

$$Q_{a}=\left( T_{e}- T_{i} \right) \times C_{p,m}\times{FR}_{i} \times\frac{1}{k}$$

*T_e_* is the effluent air temperature (in K), *T_i_* is the influent temperature (in K), *C_p,m_* is the constant pressure molar heat capacity of air (29.19 J•mol−1•K−1), *FR_i_* is the STP-corrected influent air flow rate (in mL/min). The unit conversion constant k is 1344000 mL•sec•mol-1•min-1. The output of the *Q_a_* equation (in W) was converted to kcal/hr by multiplying by 0.86042065 (kcal/hr)•W-1. Throughout, we used the thermochemical calorie (1 cal = 4.184 J), not the international steam table calorie (1 cal = 4.1868 J).

*Q_w_* was determined by the following equation:

$$Q_{w}=\frac{{FR}_{e}\times\frac{P_{vap,e}}{P_{e}}-{FR}_{i}\times\frac{P_{vap,i}}{P_{i}}}{1-\frac{P_{vap,e}}{P_{e}}}\times H_{vap}\times k$$

*FR_e_* is the STP-corrected effluent air flow rate (in mL/min), *P_vap,e_* is the water vapor pressure of the effluent air (in Pa), *P_e_* is the effluent air pressure (in Pa), *FR_i_* is the STP-corrected influent air flow rate (in mL/min), *P_vap,i_* is the water vapor pressure of the influent air (in Pa), and *P_i_* is the influent air pressure (in Pa). The unit conversion constant k is 1.34042x10-5 g•min•mL-1•s-1. The output of the *Q_w_* equation (in W) was converted to kcal/hr by multiplying by 0.86042065 (kcal/hr)•W-1.

Water vapor pressures (*P_vap,e_* and *P_vap,i_* in Pa) were calculated using the following equations, which include calculation of the saturation pressure of water by the Antoine equation:

$$P_{sat}={10}^{\left[ 8.07131-\frac{1730.63}{233.426+T} \right]}$$

$$P_{vap}=P_{sat}\times RH\times k$$

Where *T* is the air temperature (in K), and *RH* is the relative humidity (in %). The unit conversion constant k is 133.322 (in Pa•mmHg-1).

*H_vap_* is the heat of vaporization of water (in J•g-1), which was calculated in real-time by the following equation, which is a linear interpolation between (91192.5 Pa, 2265.65 J/g) and (101325 Pa, 2257.92 J/g):

$$H_{vap} = 2335.22-0.000762892\times P$$

Where *P* is the air pressure (in Pa). This value was generally close to 2260.4 J•g-1.

*Influent Air Conditioner*

Influent air was supplied under positive pressure from a laboratory supply line, with pressure regulated to adjust air flow through the system. Air was bubbled through water at room temperature (to achieve 100% relative humidity, RH), then passed through condenser columns to reduce temperature to ~5°C. Air then passed through a heated copper coil maintained at 30°C before entering the direct calorimeter. This conditioning system thereby supplied a constant influent air stream into the direct calorimetry chamber of ~30°C with a dew point of 5°C, which results in a ~19% RH. Air flow was recorded continuously using an EM1 mass flow meter.

*Respirometer*

Effluent air from chamber was sampled at 250 mL/min STP, passed through a Drierite (anhydrous CaSO4) desiccant column, and analyzed sequentially for carbon dioxide (model CD-3A, AEI) and oxygen (model S-3A/II, AEI) content. Mass flow and gas concentration data were logged using a PowerLab with associated Chart software (ADInstruments). Heat production by respirometry (in kcal/hr) was calculated using the equation derived from Lusk [[4](#_ENREF_4)]:

$$Heat=VO_{2} (1.232 RER+3.815)$$

*VO_2_* (in L/hr) was calculated as the change in O_2_ content of effluent air while a mouse was in the direct calorimetry chamber versus baseline, multiplied by the rate of effluent air flow. RER was calculated as the change in CO_2_ content divided by the change in O_2_ content.

*Calibration procedures*

Calibration of the oxygen analyzer was performed using a primary standard gas mixture containing 20.50% oxygen (Praxair; paramagnetic certification method, certified to ±0.02%). Two-point calibration of the carbon dioxide analyzer was performed using a soda lime column (resulting in 0.0000% CO2) and a primary standard gas mixture containing 5000 ppm carbon dioxide (Praxair; flame ionization with methanizer certification method, certified to ±1%). Calibrations were performed daily, before each mouse was analyzed.

Calibration of the direct calorimeter was performed by measuring total heat release from a 10.97-Ω resistor plate (including connecting wires) within the calorimetry chamber. The resistive plate was repeatedly cycled among 0.0000, 0.8432, 1.6329, and 2.4620 volts, yielding 0.0000, 0.06658, 0.2497, and 0.5676 kcal/hr (to bracket the expected ~0.15 kcal/hr RMR of a wildtype mouse) in a “with flow” (300 mL/min @ STP) condition, to simulate actual recording sessions. Linear regression analysis of the resultant calibration curve yielded the equation: Heat (in kcal/hr) = 0.02337 * voltage + 0.0004660, with an R^2^ value of 0.99992.

*Comments regarding system precision and stability*

Influent and effluent mass flows through the calorimetry chamber, and through the oxygen/carbon dioxide subsampling system (as illustrated in our previous report [[1](#_ENREF_1)]) were each determined throughout all studies using CMOSens EM1 Mass Flow Meter for Gases (0-500 mL/min) by Sensiron. According to the manufacturer, when operating near 300 mL/min (as used for the study), these mass-flow meters exhibit a repeatability [precision] of 0.9 mL/min (0.3% of reading).

The EM1 flow meter performs an internal averaging function to average 200 samples/second down to a single recorded flow rate value. Further, because our data recording rate is 1 sample/second and as a typical “sleeping” value is averaged over minimally 10 minutes of recording time, our [flow] value is time-averaged for >120,000 individual raw flow rate data points, which increases our confidence in the precision of the flow measurement. Baseline/background data for each individual mouse is recorded overnight (e.g. Σ 12 hours) before the recording session, and thus the flow rate for this single “background” value is an average of ~8,640,000 individual flow samples. Therefore the time-averaged precision will be substantially improved over the specified 0.3% rating.

The [accuracy] of EM1 flow meters is rated at 3% of the reading (in this example, with flow at ~300 mL/min, that equates to 9 mL/min). Nonetheless, with regard to the [accuracy] of the flow measurement in the calculation of RMR in the current study, this [accuracy] value is irrelevant for the following reason: RMR is calculated by determining the difference in values for each mouse compared to its own pre-recording-session baseline value. Thus, the net [accuracy] of the RMR values determined herein by both methods is essentially dependent upon the [precision] of the flow meter, not the reported instantaneous [accuracy].

*Testing period procedures*

Mice were placed into the direct calorimeter in the morning (typically between 8 and 9 AM, on a 6 AM:6 PM light cycle), and there was no food or water available inside the testing chamber as water sources confound the analysis of real-time evaporative water loss. Heat dissipation / gas exchange / core temperature data are recorded and plotted continually for the testing period, which typically lasts 4-6 hours. This time-course was chosen because it is required to achieve a stable sleep epoch (evident by stable plateau in all endpoints). Therefore the animal is fasted roughly 4-6 hours at the time of data capture for inter-group / inter-method comparisons, which corresponds to a commonly used fasting period (5 hrs) for measures of glycemic control in mice.

Supplement References

1. Burnett, C.M. and Grobe, J.L., 2013. Direct calorimetry identifies deficiencies in respirometry for the determination of resting metabolic rate in C57Bl/6 and FVB mice. Am J Physiol Endocrinol Metab 305:E916-924.

2. Lighton, J.R.B., 2008. Measuring metabolic rates. New York:Oxford University Press.

3. McLean, J.A. and Tobin, G., 1988. Animal and human calorimetry. New York:Cambridge University Press.

4. Lusk, G., 1928. The elements of the science of nutrition. 4th edition, Philadelphia, PA:W.B. Saunders Company.
